# Using anatomically defined regions-of-interest to adjust for head-size and probe alignment in functional near-infrared spectroscopy

**DOI:** 10.1117/1.NPh.7.3.035008

**Published:** 2020-09-23

**Authors:** Xuetong Zhai, Hendrik Santosa, Theodore J. Huppert

**Affiliations:** a University of Pittsburgh, Department of Bioengineering, Pittsburgh, Pennsylvania, United States; b University of Pittsburgh, Department of Radiology, Pittsburgh, Pennsylvania, United States; c University of Pittsburgh, Clinical Science Translational Institute, and Center for the Neural Basis of Cognition, Department of Electrical and Computer Engineering, Department of Bioengineering, Pittsburgh, Pennsylvania, United States

**Keywords:** functional near-infrared spectroscopy, statistical analysis, functional brain imaging

## Abstract

**Significance:** Functional near-infrared spectroscopy (fNIRS) uses surface-placed light sources and detectors to record underlying changes in the brain due to fluctuations in hemoglobin levels and oxygenation. Since these measurements are recorded from the surface of the scalp, the mapping from underlying regions-of-interest (ROIs) in the brain space to the fNIRS channel space measurements depends on the registration of the sensors, the anatomy of the head/brain, and the sensitivity of these diffuse measurements through the tissue. However, small displacements in the probe position can change the distribution of recorded brain activity across the fNIRS measurements.

**Aim:** We propose an approach using either individual or atlas-based brain-space anatomical information to define ROI-based statistical hypotheses to test the null involvement of specific regions, which allows us to test the analogous ROI across subjects while adjusting for fNIRS probe placement and sensitivity differences due to head size variations without a localizer task.

**Approach:** We use the optical forward model to project the underlying brain-space ROI into a tapered contrast vector, which defines the relative weighting of the fNIRS channels contributing to the ROI and allows us to test the null hypothesis of no brain activity in this region during a functional task. We demonstrate this method through simulation and compare the sensitivity-specificity of this approach to other conventional methods.

**Results:** We examine the performance of this method in the scenario where head size and probe registration are both an accurately known parameters and where this is subject to unknown experimental errors. This method is compared with the performance of the conventional method using 364 different simulation parameter combinations.

**Conclusion:** The proposed method is always recommended in ROI-based analysis, since it significantly improves the analysis performance without a localizer task, wherever the fNIRS probe registration is known or unknown.

## 1 Introduction

Functional near-infrared spectroscopy (fNIRS) is a noninvasive neuroimaging technique that uses low levels of red to near-infrared light to measure changes in the optical absorption due to hemoglobin in the brain. Typically, light is sent into the tissue from source positions on the scalp. This light diffuses through the tissue, and a small fraction of the light is detected at a discrete set of optical detector positions placed several centimeters from the originating source position. These channel-space measurements are sensitive to changes in the optical properties of the tissue along this diffuse volume between the light source and detector. During an evoked functional task, the changes in blood flow and oxygenation in the brain result in fluctuations in optical absorption due to hemoglobin in this local region, and this gives rise to changes in the fNIRS measurements in the source–detector pairs (channels) crossing this region. Using a grid of these optical source and detector positions embedded in a head probe, functional brain activity can be recorded from regions of the surface of the brain’s cortex. Over the last three decades, fNIRS has been used in a variety of different brain imaging studies and populations (reviewed in Refs. 1–4). In particular, the ability to noninvasively record brain activity without participant immobilization or a specialized dedicated scanner environment (cf. magnetic resonance imaging; MRI) makes this technique well suited for studies in pediatric populations (reviewed in Refs. 1 and 5–7).

A challenge of fNIRS measurements, however, is group-level registration of these signal changes from these sparse surface-based measurements. Small displacements in the probe position relative to the underlying brain region can change the distribution of recorded brain activity across the fNIRS measurements. This is particularly problematic in cross-sectional or longitudinal studies of child development, where the head size varies between sessions. Moreover, these measurements are also sensitive to intersubject differences in head anatomy, such as skull thickness and depth of the brain relative to the skin’s surface. Thus, this uncertainty increases variance across measurement sessions and reduces statistical effects sizes. This also makes studies of brain activation changes with child age and development difficult.

An alternative approach to this would be to use an individual’s response to a “localizer” functional task to define consistent underlying brain regions across participants. While this data-driven approach makes fewer assumptions than atlas-based models (as will be detailed in this work) to define regions-of-interest (ROIs), this method requires the ability to robustly measure a specific localizer task response for a given brain region in each subject. This is not always possible since not all brain regions can be specifically and uniquely defined by localizer tasks, which may involve multiple regions of the brain. In addition, single-subject statistics are often not reliable enough to define individualized regions for many tasks or subject populations. Thus, while the use of a localizer task is recommended when possible, more generalizable solutions are also needed.

Methods for the spatial registration of the fNIRS head cap and measurement channels with respect to the brain have been described in previous work.^8^^–^^11^ Although not always possible or practical, fNIRS investigators often record this information with either a three-dimensional (3-D) camera and registration system (e.g., Ref. 12) or simply using a tape measure to record head-size and potentially the location of the fNIRS sensors relative to the international 10-20 system. However, although this registration information is recorded as part of fNIRS experimental best practices by many labs, there has been very little development of quantitative methods to actually quantitatively use this information within fNIRS analysis.

In this work, we propose a new approach to quantitatively incorporate head- size, probe registration, and/or individual anatomical information to define ROIs for fNIRS analysis. In this proposed method, we make use of the optical “forward model,” which describes the sensitivity of a particular fNIRS source–detector pair to the underlying brain regions based on the diffusion/transport of light in the tissue. The optical forward model is used to create a testable null hypothesis about the involvement of a particular region of the brain using a weighted average of the measurement channels. For example, based on the registration of the fNIRS probe, brain activity from a particular Brodmann area^13^ region would be expected to be highest on a specific fNIRS channel with tapered responses to nearby channels. Using this tapered spatial distribution of expected signal changes allows us to create a statistical model of what the fNIRS data would be expected to look like if this region was active in the task. Likewise, this creates a testable null hypothesis — if this signal change in this region was not different from zero then a spatially weighted average over this particular set of channels would also be not differ from zero. If the weighted average over these channels was nonzero, then we can reject this null hypothesis. The rejection of this null hypothesis means that we cannot rule out that this region was active during the task, but does not actually imply that the signal definitely came from this region as opposed to a nearby or smaller region of the brain, which was also sampled by this set of channels. Nonetheless, the interpretation of such a result would be that the recorded brain signals are consistent with that particular region’s involvement in the task.

Since this method utilizes the optical forward model, it provides means to adjust the null hypothesis based on head size, probe registration, and/or individual anatomy. For example, the expected projection of a region such as dorsal-lateral prefrontal cortex might be higher on more lateral fNIRS channels in a subject with a smaller head size compared to a subject with a larger head using the same fNIRS probe and spacings. In most cases, particularly in studies of children, knowledge of individual brain anatomy (e.g., via MRI) is not practical, but measurements of head size, placement of the probe relative to 10-20 locations, or 3-D positioning cameras are often recorded and can be used in this proposed method. In addition, this approach does not require a separate localizer task condition to define the ROI. While the activation maps from a separate localizer task provide an objective way to define an ROI on an individual subject basis, this approach is not always practical. In addition, if the localizer task is not exceptionally strong/statistical, there will be uncertainty in the region definitions.

In this paper, we describe the theory behind our approach to use individualized tapered weights to define the statistical contrast for ROIs and compare the use of tapered and uniform weighted models. We also examine the effect of small errors in the probe registration on the model performance to examine the method under realistic conditions.

## 2 Theory

### 2.1 Analysis of fNIRS Data

Functional NIRS data are recorded as changes in the light from a source position incident on a detector position (e.g., transmitted between a source–detector pair) as a function of time. These signals are first converted to changes in optical density (optical absorption) over time as given as

$$\Delta OD(t)=-\log[\frac{I(t)}{I_{0}}],$$ (1)

where I(t) is the intensity of the signal recorded and $I_{0}$ is the reference signal intensity at baseline (usually taken as the mean of the signal over the scan). The optical density changes at wavelength λ are then transformed into estimates of oxy- and deoxyhemoglobin (HbO/HbR) changes using the modified Beer–Lambert law^14^

$$OD^{\lambda }=l\cdot {\mathrm{DPF}}^{\lambda }[\varepsilon_{\mathrm{HbO}}^{\lambda }(\Delta [\mathrm{HbO}])+\varepsilon_{\mathrm{HbR}}^{\lambda }(\Delta [\mathrm{HbR}])],$$ (2)

where l is the source–detector distance and DPF is the differential pathlength. Δ[HbX] is the change in molar concentration and $\varepsilon_{\mathrm{HbX}}$ is the molar extinction coefficient, where HbX represents either HbO or HbR for oxy- and deoxyhemoglobin, respectively.

In most evoked fNIRS studies, a task(s) is repeatedly preformed while recording the fNIRS signals. A first-level statistical model (subject-level statistics) is then used to examine changes in the fNIRS signals during the task.^15^^–^^17^ More formally, the linear regression model is described by the equation

Y=Xβ+ε, (3)

where X is the design matrix of the modeled hemodynamic response encoding the timing of stimulus events and β is the coefficient (weight) of that stimulus condition for that source–detector channel. This statistical model can be either a block average, deconvolution, or canonical hemodynamic response method (see Ref. 17), which results in an estimated statistical parameter (typically and herein termed β) and its uncertainty across the spatial channels (herein termed ${\mathrm{cov}}_{\beta }$). Specifically, in the case of block averaging or deconvolution, β would be the parameter of interest (mean over a time window, maximum, etc.) computed from the estimated response. In the case of a canonical linear model (or something similar), then β would be the estimated coefficient for the regression model. In general, $\beta_{i}$, the i’th element of β, is just a statistical parameter associated with the i’th spatial fNIRS measurement channel upon which we are basing the hypothesis test (e.g., $\beta_{i}$ differs from zero). The spatial covariance of this parameter is denoted as ${\mathrm{cov}}_{\beta }$.

Based on the estimate of the parameters and their uncertainties over the multiple channels in a fNIRS probe, the calculation of a Students t-statistic for an ROI is given as

$$t=\frac{c\cdot \beta }{\sqrt{c^{T}\cdot {\mathrm{cov}}_{\beta }\cdot c}}.$$ (4)

The contrast vector (c) denotes the weights given to channels being averaged. In this expression, β is a vector denoting the statistical parameter for each spatial channel of the fNIRS probe. Note, this formalism allows for multiple task conditions for each channel, but for simplicity of explanation, we will assume there is only one task-associated parameter ($\beta_{i}$) per measurement channel. In the case of multiple task conditions, the contrast vector is the Kronecker product (⊗) of the spatial contrast vector and the per-task-condition contrast vector and the β and ${\mathrm{cov}}_{\beta }$ terms contain all tasks and spatial channels.

The statistic defined by Eq. (4) can be used to test the following null hypothesis:

$$H_{0}:\overset{N}{\underset{i=1}{\sum }}c_{i}\cdot \beta_{i}=c\cdot \beta =0,$$ (5)

where N is the total number of channels. For example, $c={\left[\frac{1}{3},\frac{1}{3},\frac{1}{3},0,0,0\right]}^{T}$ would be used to average the values of the first three (of six) spatial channels with uniform weights. This contrast vector (c) encodes the null hypothesis being tested, which, in this example, is that the mean of the first three channels is not different from zero. This is the same expression as used to compute contrast between tasks for a single spatial channel (e.g., task A verses task B) where the covariance is described between the conditions (e.g., from linear regression analysis) (see Ref. 18).

### 2.2 Proposed Method

A statistically significant $\beta_{i}$ (different from 0) that represents the signal in the i’th channel has a strong relationship with the modeled hemodynamic response and consequently indicates the area of the cortex efficiently covered by this channel is not inactive. The statistical significance of a linear combination of βs implies the activity of the area covered by the corresponding channels, and the coefficients (weights) of the linear combination can determine the shape of the area. In the previously (Sec. 2.1) shown example, the entire test area consists of the regions covered by the first three channels with equal weights. However, the ROI in an experiment is rarely a combination of areas equally covered by several channels, especially the predefined anatomical areas, e.g., Brodmann areas,^13^ since the sensitivity to a given area is maximized in the nearest channel and decreases with the distancing from the channel. Thus, we propose that the contrast vector (c) in Eq. (4) can be used to test the null hypothesis of the noninvolvement of specific underlying regions of the brain. Specifically, instead of using uniform weights to sum over a specific set of channels as used in the previous example, we propose to use a tapered contrast vector that peaks on the spatial channel most expected to be active in the hypothesis and lowers based on the relative sensitivity of other channels to this same region. Examining Eq. (4), we note that the numerator in this expression is the inner product of the c and β vectors. This inner product is maximized when the two vectors point in the same direction, which implies that the t-statistic will be largest when the spatial distribution of the c vector matches the expected spatial distribution of the brain activity. Ergo, if the brain activity came from a particular region such as BA-46 defined by the Brodmann areas,^13^ then the t-statistic will be maximized when the contrast vector has the tapered spatial distribution consistent with the fNIRS probe placement relative to this region. Comparing to the conventionally used uniform weights, the tapered weights increase the contribution of the expected region and decrease that of the noise from other areas. Thus, this is the most conservative test of the null hypothesis. This approach allows us to first pose specific null hypothesis tests about underlying regions of the brain. For example, if BA-46 were not active in the task, then the specific spatial weighing of channels would not differ from zero. While the rejection of this null hypothesis (e.g., finding that the value of the ROI average differs from zero) implies that we cannot rule out that (e.g.,) BA-46 was involved in the task, this however, does not mean that BA-46 specifically was involved rather than some other nearby region. Second, this formalism allows us to statistically test the involvement of different regions. For example, using two separate contrast vectors we can test if the brain activity was more consistent with (e.g.,) BA-46 or BA-45^13^ by statistically comparing those two t-statistics.

We propose that this tapered spatial weighting of channels is based on the optical forward model, the registration of the fNIRS probe, and the underlying regional parcellations of the cortex. The optical forward model [Eq. (2)] defines the sensitivity of the measurements in channel space to underlying changes in the brain space. This model is calculated by estimation or simulation of the diffusion of light through the tissue (e.g., Refs. 19 and 20). The optical forward model provides an estimate of the expected signal changes for the fNIRS measurement geometry given by the expression

$$Y=L\mu_{\text{volume}},$$ (6)

where Y is the measurement for a specific fNIRS probe, L is the forward model relating that probe layout and registration to the underlying head/brain, and $\mu_{\text{volume}}$ is the underlying change in optical absorption in the volume. Based on the registration of the fNIRS probe to an anatomical atlas or individual anatomy (if available), the expected relative sensitivity of each fNIRS source-to-detector channel can be estimated from the location and depth of anatomically defined regions through the optical forward model.

To test for statistical activity from specific anatomically based ROI, we can use the optical forward model and Eq. (6) to define the hypothesis of what the activity pattern in channel-space should look like based on the location in volume (brain) space. In other words, to form the null hypothesis testing for activity in a specific ROI, the contrast vector is given as

$$c_{\mathrm{ROI}}=L\cdot {\text{Mask}}_{\mathrm{ROI}}(r),$$ (7)

where the mask vector for a specific ROI is defined by Eq. (8), in which r represents each point of cerebral cortex

$${\text{Mask}}_{\mathrm{ROI}}(r)=\{\begin{array}{cc} \begin{array}{c} 1\\ 0 \end{array} & \begin{array}{c} \mathrm{if}\;r\in \mathrm{ROI}\\ \text{otherwise} \end{array} \end{array}.$$ (8)

To generalize this method for multiple conditions comparison, the contrast vector used in Eq. (4) can be replaced as

$$c=c_{\mathrm{ROI}}⊗c_{\mathrm{COND}}.$$ (9)

Here, $c_{\mathrm{COND}}$ is the contrast vector for the pooling of conditions. Then, a t-test can be performed using the statistic defined by Eq. (4) with the proposed contrast vector given by Eq. (9).

### 2.3 Example

**Fig. 1:**
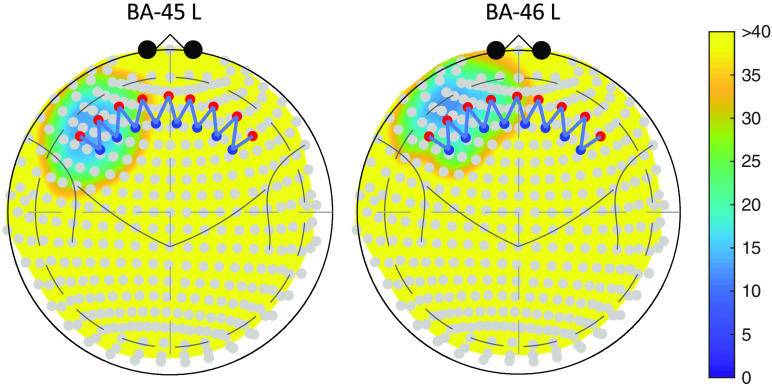
The position of the two ROIs: BA-45 L and BA-46 L in Colin27 atlas with a head circumference of 420 mm. The color map represents the depth from each node in the ROI to the head surface. Yellow area indicates a depth greater than 40 mm, which is unreachable by the light.

**Fig. 2:**
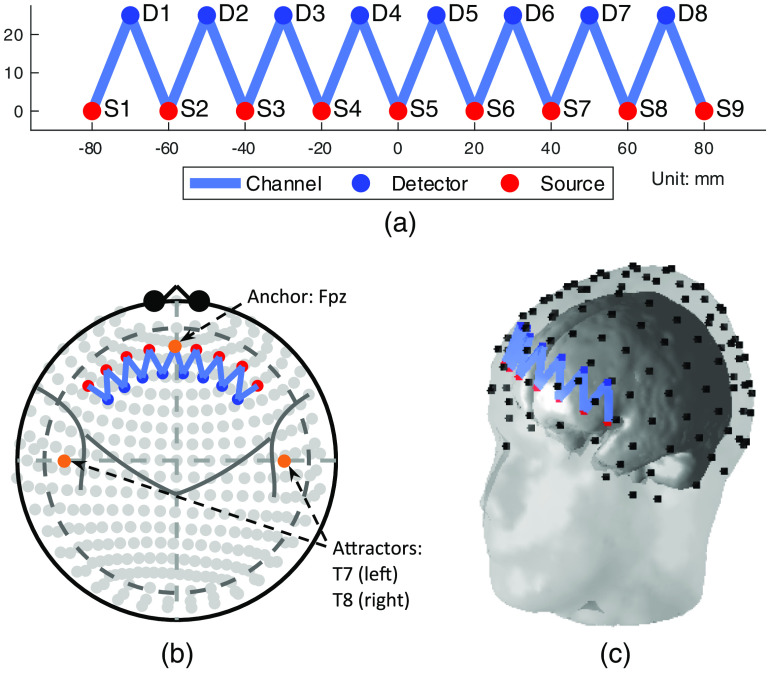
The topology of the low-density probe used in the simulation: (a) The 2-D layout, (b) the registered probe with the 10-20 International System, and (c) the registered 3-D probe geometry. A head with a 420-mm circumference is used in (b) and (c).

In this section, we demonstrate the process of contrast vector calculation for a specific ROI and the analysis with the contras vector. Suppose we are interested in whether BA-45 left or BA-46 left is involved in an experiment. In this example, we would also like to test the difference between the activities of the two ROIs. Figure 1 shows the two regions in Colin27 atlas^21^ and an example probe registered to the 10-20 system. See Sec. 3.1.1 and Fig. 2 for the details of the probe.

Based on the registration of this probe and the labeled parcellations of the brain (in this case the Taliarach-deamon^22^ parcellation of the Colin27 atlas^21^), we can construct a spatially tapered contrast vector using Eqs. (6)–(8). Table 1 shows the weights of each channel in the contrast vector. The first two columns are the weights for the two ROIs—BA-45 L and BA-46 L, respectively, and the third column contains the weights to test the difference between them, which are obtained by simply subtracting the second from the first column. As a comparison, the conventionally used uniform weights are also listed in the table, which are obtained by assigning equal weights to the nearest four channels to each region.

**Table 1:** Each row of the table contains the weights of the channels for the two ROIs and the difference between them. The weights are calculating using Eqs. (6)–(8). It can be seen that a nearer channel has a larger weight. S and D represent source and detector, respectively, whose indices can be found in Fig. 2. Both the proposed tapered and conventional uniform weights are listed. Note that the remaining channels are omitted from visualization in this table because of their small weights.

fNIRS channel	Weights of channels for ROI					
	BA-45 L		BA-46 L		{BA-45 L}−{BA-46 L}	
	Proposed	Uniform	Proposed	Uniform	Proposed	Uniform
S 1 : D 1	0.193	0.25	0.027	0	0.166	0.25
S 2 : D 1	0.209	0.25	0.121	0	0.089	0.25
S 2 : D 2	0.314	0.25	0.224	0.25	0.09	0
S 3 : D 2	0.213	0.25	0.23	0.25	−0.017	0
S 3 : D 3	0.053	0	0.137	0.25	−0.084	−0.25
S 4 : D 3	0.014	0	0.13	0.25	−0.116	−0.25
S 4 : D 4	0.003	0	0.095	0	−0.092	0
S 5 : D 4	0.001	0	0.026	0	−0.026	0
S 5 : D 5	0	0	0.007	0	−0.007	0

## 3 Methods

In this paper, we compare the proposed tapered contrast vector method to the conventional analysis methods using simulation data. We compare two different approaches to computing the ROI contrast: (1) in the uniform weighting scheme, the four channels closest to the underlying ROI are selected, and these channels are given a uniform weighting in the contrast vector; (2) in the tapered weighting scheme, the forward model is used to compute the relative contribution of the region to each channel based on head size and probe registration. For the both weighting schemes, we additionally examined the case in which this registration information is known accurately and where it is subject to experimental measurement errors, which is described in Sec. 3.1.3. Here, the weights are calculated based on the head size and probe registration of each subject. The unknown condition was examined to mimic the realistic case of experimental error in the registration in which the weights are calculated based on the average head size regardless of probe registration error and individual head-size differences. In each iteration of the simulation, we generate a group set of fNIRS data containing five subjects with the same stimulus within the region of the brain and perform group-level channel-space analyses using contrast vectors containing weights of different channels using both the proposed tapered weights for all channels and uniform weights for the nearest channels with both assumptions that the probe registration is known and unknown. In total, there are four analysis conditions, tapered known, tapered unknown, uniform known, and uniform unknown, in each simulation iteration. The analysis results of the two methods are investigated via receiver operating characteristic (ROC) and compared to each other.

### 3.1 Probe Configurations

In this work, two types of probe configuration: low-density and high-density probes, are used for simulations. While the low-density style of probe configuration is much more frequently used in fNIRS studies due to practical reasons, this style of probe has “blind-spots” due to regions of low sensitivity to underlying brain activity.^23^ Thus, low-density probes are more sensitive to displacements in the registration of the fNIRS probe and/or variations in subject head size. In contrast, high-density fNIRS probes (e.g., Ref. 23) have more uniform spatial sensitivity and fewer blind spots, but are more complex to record from and are only supported by a few instrument manufacturers.

#### 3.1.1 Low-density probe

The low-density probe contains nine light sources and eight detectors. The distance between source and detector alignments is 25 mm. The optical density is only measured between the nearest source–detector pairs. Hence, there are 16×2 (two wavelengths, 16 channels for HbO, and the other 16 for HbR) channels defined in the low-density probe. An equal weight, ¼, is assigned to each of the nearest four channels to construct the uniform contrast. Figure 2(a) shows the two-dimensional (2-D) layout of the probe.

The registration of the probe is defined by an anchor and three attractor positions on the probe. Similar to the use of these terms in the AtlasViewer program,^8^ anchors and attractor positions help to register the fNIRS probe onto the 10-20 coordinate system. In the Brain AnalyzIR toolbox,^18^ an anchor forcibly fixes a point of the probe layout [Fig. 2(a)] on the 10-20 system placement. In this case, the origin of the probe (0, 0) in the 2-D layout is anchored to the 10-20 site Fpz. An attractor provides directional information to the probe. Here, three attractors are placed at positions (±200, 0) and (0, 100) in the 2-D layout and are attached to T7, T8, and Cz, respectively, which define three forces pulling the probe along negative/positive X axis and positive Y axis pointing to T7, T8, and Cz. The registration algorithm uses an iterative least-squares minimization algorithm based on the optimal source–detector pair spacings and the location of the anchor/attractor points. Attractor points are used to construct unit vectors to provide direction, which are updated with every iteration of the algorithm. The registered probe used in this example is shown in Figs. 2(b) and 2(c) using 10-20 (Mercator) projection and 3-D geometry on an example head with 420-mm circumference.

#### 3.1.2 High-density probe

**Fig. 3:**
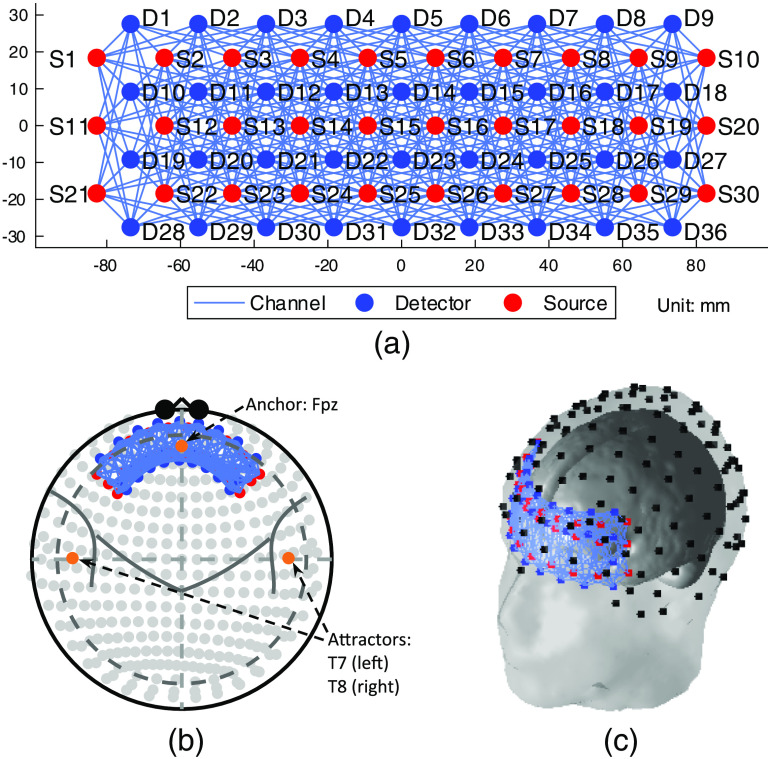
The topology of the high-density probe used in the simulation: (a) the 2-D layout, (b) the registered probe with the 10-20 International System, and (c) the registered 3-D probe geometry. A head with a 420-mm circumference is used in (b) and (c).

The high-density probe used in this work is suggested by Zeff et al.^23^ Measurements are made between the first-, second-, third-, and fourth-nearest neighboring source–detector pairs, the separations of which are 13, 30, 40, and 48 mm,^23^ respectively. The distance between two neighboring sources or detectors can be consequently calculated as $13\sqrt{2}=18.385$mm. Instead of the 24 sources and 28 detectors used in the previous study,^23^ we added six sources and detectors for covering a similar length of area with the low-density probe used in Sec. 3.1.1. Thus, our high-density probe contains 30 sources and 36 detectors, which form 460×2 channels in total. To be comparable with the low-density probe, a quarter of the channels (115/460) are used to calculate the uniform contrast vector with equal weights. The 2-D layout of the high-density is shown in Fig. 3(a).

The anchor and attractors used in the high-density probe registration are same as those for the low-density probe defined in Sec. 3.1.1. Figures 3(b) and 3(c) present the high-density probe registration in the 10-20 system and 3-D geometry on a head with 420-mm circumference.

#### 3.1.3 Probe registration with head size and displacement consideration

In this work, in addition to examining the ideal case in which the fNIRS probe registration and head/brain size are perfectly known, we also examined the realistic case in which these parameters had unknown errors associated with them. In particular, it is conceivable that using error-prone prior (mis-)registration information could actually hurt the accuracy of the analysis methods and we wished to examine the sensitivity of the method to these errors. Figures 2(b) and 3(b) show the ideal situation of the probe registration in which the head size is known, and the anchor and attractors are placed without any errors. However, in most practical fNIRS experiments, the subjects’ head circumferences are not recorded, and placement errors are unavoidable. We use random head sizes and probe registration errors in this study. In our simulation, the head circumferences are generated from a normal distribution with a mean of 420 mm and a standard deviation of 50 mm. The lower and upper 2.5% quantiles of this distribution are 318.08 and 521.92 mm. This was chosen such that the simulated head circumference falls into the head-size range of 0 to 36 months infants^24^ with a 95% probability. To simulate the registration error, the anchor and attractors are placed at a position that deviates from the original position by a random distance. The displacements along X and Y axes are both randomly generated from a normal distribution with a zero mean and a standard deviation of 10 mm. Since the upper 2.5% quantile of this distribution is 19.60 mm, Fpz falls into a square with a center at (0, 0) of the probe layout and an edge of 39.20 mm with a probability of 1−5%×5%=99.75%. Similarly, in 95% cases, the X and Y axes of the probe are pulled to T7/T8 and Cz with angle errors in the ranges of ±5.60 deg [arctan(19.60/200)] and ±11.01 deg [arctan(19.60/100)], respectively.

**Fig. 4:**
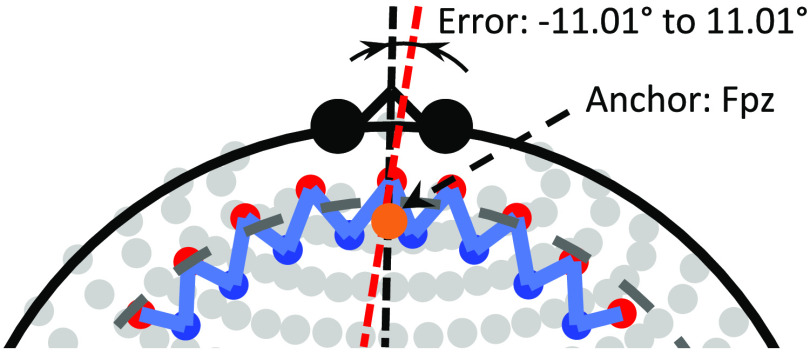
An example of probe registration with displacement error. Compared to Fig. 2(b), the registered probe is asymmetric. The red dashed line is the centerline of the probe, which does not coincide with the brain centerline (the vertical black dashed line). As explained above, the middle light source, S5 at (0, 0), deviates from the anchor point Fpz, and the angle between the two centerlines is in the range of (−11.01 deg, 11.01 deg). The left and right parts of the probe may independently rotate around the probe centerline (red dashed line) by an angle between −5.60 deg and 5.60 deg.

Figure 4 is an example of probe registration with a larger head circumference (485 mm) and random error. Comparing to Fig. 2(b), the middle light source of the probe is not placed exactly on the anchor point, which is caused by the placement error. It can also be seen that there is an angle between the two centerlines in a range of ±11.01 deg. The left and right parts of the probe may independently rotate around the centerline of the rotated probe by an angle within ±5.60 deg. Therefore, even in the worst case, each of the left and right parts is unlikely to deviate from the ideal position more than 16.61 deg (the probability exceeding this value is 5%×2.5%=0.125%).

### 3.2 Region-of-Interest Selection

As mentioned in Sec. 2.2, two types of analysis are performed in this work—the involvement of a specific ROI and the difference between the activities of two ROIs, which can be described as two statistical tests: (1) test if the hemodynamic response within a specific ROI is significantly different from zero and (2) test if there is a statistically significant difference between the hemodynamic responses in two nearby ROIs. For the single ROI analysis, the size of the region is considered as a factor in the simulation. In addition, the distance between the two nearby ROIs is taken into account as another factor in the ROI difference analysis. The selection of ROIs for these two types of analyses is described in the following two sections.

#### 3.2.1 Single ROI analysis

**Fig. 5:**
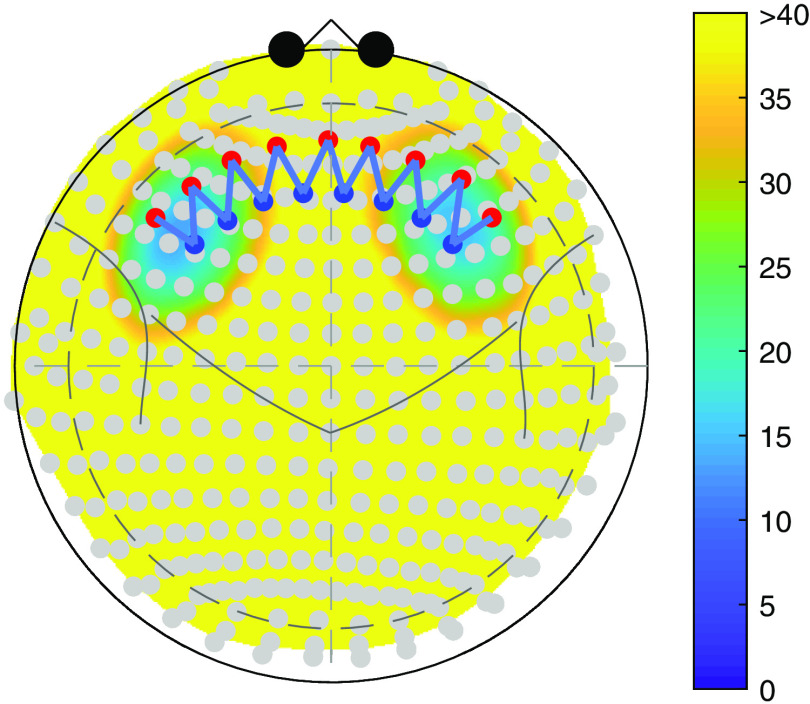
An example of ROI selection. The color map represents the depth from each node in the ROI to the head surface. The yellow area indicates a depth greater than 40 mm, which is unreachable by the light. The stimulation is generated within the ROI in the left hemisphere while the right one is used as the null region.

The ROI used in this work is created using a spherical surface with a center at a node (brain coordinate) from Colin27 atlas^21^ and a specified radius. All nodes included within the sphere define the ROI. Because of the symmetry in the cerebral cortex, we only select ROIs and generate stimulation in the left cerebral hemisphere while the mirrored ROIs in the right hemisphere are used as the null regions (containing noise only) to evaluate the false positive rate (FPR) of the analysis. Figure 5 is an example of ROI selection.

Note that the distance between the center node of each ROI and the nearest optode must not exceed the specified radius of the ROI so that the ROI can be reasonably covered by the probe. Another consideration for ROI selection is the head size. To perform a reasonable group-level analysis, the relative size of each ROI to the entire cerebral cortex must be comparable between subjects. In this study, the nodes included in an ROI are selected using the standard Colin27 atlas, then the coordinates of the nodes are scaled according to the head circumference ratio.

#### 3.2.2 Statistical testing between two ROIs

**Fig. 6:**
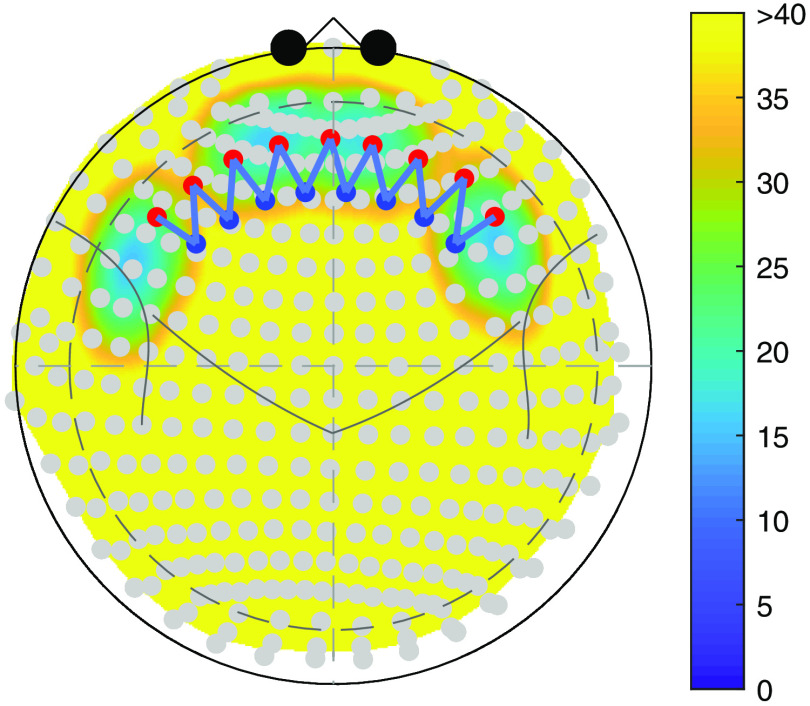
An example of ROI selection for region difference analysis. The legend is same as that in Fig. 5. Note that it looks like the ROIs in the left and right hemispheres overlap each other, but they do not actually because of the existence of a cerebral fissure.

The selection process for ROI difference analysis is similar to that for single ROI analysis but involves the distance between two ROIs as a new factor. Figure 6 is an example of ROI selection for difference analysis. For the same reason, which is described in Sec. 3.2.1, we only generate stimulation within one of the two ROIs in the left hemisphere and calculate the difference. The two mirrored ROIs in the right hemisphere are considered as null region, the difference of which is used to evaluate the FPR.

One more factor, the distance between the two ROIs, is taken into consideration in addition to head size and ROI radius. We first select an ROI using the method described in Sec. 3.2.1 and generate stimulation within it, then find out all nodes that deviate the center of the ROI by a specified distance, from which the second ROI used to calculate the hemodynamic difference is randomly selected.

### 3.3 Stimulus Generation

The fNIRS data are simulated by adding stimulation on autoregressive noise. The time difference between two neighboring stimuli is exponentially distributed. The hemodynamic response to the stimulus is simulated using the canonical hemodynamic response function. In brief, simulated “brain” activity within the ROI (true positive) is computed and projected to fNIRS channel/measurement space via the optical forward model. The contralateral ROI is used to define the true negative region for ROC analysis. Additive autoregressive noise is added to all channels at an SNR level of 1. The details are described in Ref. 18.

### 3.4 ROC Analysis

With the p-value reported from the t-test [Eq. (4)], we calculate the FPR and true positive rate using (1−p-value) as the threshold, since smaller p-value indicates more significant HbO and HbR changes. The ROC curve can then be constructed, and the area under the curve (AUC) is the probability that the hemodynamic responses within the stimulus-containing ROIs are more significant (with a smaller p-value) than that in the null ROIs. Thus, AUC is utilized as the indicator for analysis performance evaluation. To determine whether a method is significantly better than another, the null hypothesis that their AUCs are equal is tested. The z-statistic is defined as

$$z=\frac{\left|{\mathrm{AUC}}_{1}-{\mathrm{AUC}}_{2}\right|}{se({\mathrm{AUC}}_{1}-{\mathrm{AUC}}_{2})},$$

where se() is the standard error. The standard error of the AUC difference is estimated using DeLong’s method.^25^ The p-value for the abovementioned null hypothesis can finally be reported.

An appropriate statistical model should give evenly distributed p-values when the null hypothesis is true, i.e., p-values smaller than a threshold (commonly called type-I error control, α) will be considered as false positives, and the FPR is the empirical type-I error rate. Thus, we check the relationship between the empirical FPR and the model-reported p-value. In an ideal situation, they are equal and the plot of FPR versus p-value is the diagonal of the plotting square. Otherwise, the type-I error rate is over- or underestimated.

### 3.5 Summary of Simulation

The probe registration, noise and stimulus generation, and data analysis are implemented using Brain AnalyzIR toolbox.^18^ A total of 20 individual ROIs were selected. For each ROI, the radius of the region was examined from 10 to 36 mm by 2-mm steps (14 values in total). This yielded a total of 280 regions, which were used to generate simulated activity for the five subjects for the group analysis with randomly selected head sizes. In the case of simulations with additional registration error, uncertainty was added between the probe registration and forward model used to generate the data and the one used in the analysis. Each group simulation for each ROI was repeated 100 times, for a total of 28,000 simulations. For examining the statistical test between two ROIs, a second ROI of the same radius was added at a distance between 20 and 80 mm at 5-mm steps (13 values in total) for each of the simulations (364,000 total simulations for each of the two probe types). For each simulation parameter combination, the analyses are, respectively, performed with two assumptions: (1) head size and probe registration error are known, where the contrast vectors are calculated based on the actual registered probe, (2) head size and probe registration error are unknown, where the contrast vectors are calculated based on the probe registered to the average head size (420-mm circumference) without registration error.

### 3.6 Implementation in the Brain AnalyzIR (NIRS-Toolbox)

The calculation of the contrast vector for a given ROI has already been implemented in the Brain AnalyzIR toolbox.^18^ This is an open-source analysis toolbox written in MATLAB^®^ for fNIRS. The main components in the implementation are described in this section.

#### 3.6.1 Forward model

The AnalyzIR toolbox includes interfaces to several third-party optical forward model solvers including NIRFAST,^20^^,^^26^ mesh-based Monte Carlo (MMC^27^^,^^28^), and Monte Carlo Extreme (MCX^29^^,^^30^). This code allows construction and import of individual head models from anatomical MRI volumes to generate subject-specific optical forward models and registration. These solvers can be used with either atlas-based or individual head models to generate this optical forward model. However, since the computation of multiple optical forward models is often time consuming, and furthermore this level of anatomical modeling is often not available for all subjects (e.g., pediatric fNIRS studies), the default options in the AnalyzIR toolbox make use of a presegmented head model derived from the Colin-27 atlas.^21^ Furthermore, to achieve fast computation of the sensitivity of a particular fNIRS channel to the underlying brain region, a simplified optical forward model is approximated using the closed-form solution for the semi-infinite homogeneous slab geometry^31^ to compute a particular two-point Green’s function solution to the diffusion model (e.g., the relationship of light traveling from a point on the surface to a point in the volume). The sensitivity of a source–detector pair is then computed as the three-point Green’s function combining two obliquely oriented slab-based two-point functions. This approximation of the optical forward model (termed the ApproxSlab forward model in the toolbox) was found to give a reasonable approximation compared to formal solutions using finite element or Monte Carlo methods, particularly given the existing approximations and errors associated with the use of the Colin-27 atlas. We note also that the Brain AnalyzIR toolbox does support the use of these proper finite element or Monte Carlo solvers to compute a more accurate forward solution, but as mentioned, due to the computational time involved (several minutes per contrast vector compared to a few hundred milliseconds for the ApproxSlab model), the default in the toolbox is to use this approximate solution. All results in this work used this approximate solution.

In addition, to avoid the complexities of multivariate statistical testing between oxy- and deoxyhemoglobin and multiple optical wavelengths inherent to the Beer–Lambert law, we approximate the forward model using only a single wavelength (default at 808 nm), which allows us to compute the spatially tapered contrast weight that can be applied to the statistical parameters (β) defined in oxy- or deoxyhemoglobin. Note that the tapered contrast vector used in the ROI definitions is normalized such that the value of the extinction coefficient for oxy- or deoxyhemoglobin at that wavelength is irrelevant to the calculation.

#### 3.6.2 Labeling of regions

As mentioned previously, Colin27 atlas is used as the default anatomical model for probe registration in this work. To identify the voxels contained in a specific ROI, a parcellation of the anatomical model is required. Considering the generality, random self-defined ROIs are used in this study rather than the predefined Brodmann areas.^13^ Thus, instead of the Broadmann labeling,^13^ here the Talairach Daemon^32^ defines the ROIs, which gives a high-resolution parcellation of the brain and allows us to define high-resolution ROIs.

#### 3.6.3 Resizing of Colin27 atlas

Since the effect of the head size is investigated in this study, the anatomical model needs to be scaled for different head circumferences and dimensions. In the AnalyzIR toolbox, the atlas head size can be rescaled based on the experimental measurements of the head circumference, nasion-inion (Nasion→Cz→Inion) and left/right periocular point (LPA→Cz→RPA) distances over the top of the head where the head circumference is computed 10% above the contour of nasion—right preauricular point (RPA)—inion—left preauricular point (LPA)—nasion. These three measurements uniquely define the resizing of the head as an ovoid shape to match each subject. Alternatively, when only one of these three measurements is available (e.g., head circumference only), the head can be resized proportionately keeping the ratios of the major and minor axes of ovoid fixed. In this case, for a given head size, we calculate the ratio of the given head circumference to the standard model’s, then resize the atlas by multiplying the Talairach coordinates^33^^,^^34^ of every point by the ratio of their head circumferences. As a result, the portion of a specific ROI will be the same in the scaled atlas. In the Brain AnalyzIR toolbox, registration of an fNIRS head cap to a brain model is done in two steps to (i) first register the cap to the ovoid (spherical) 10-20 coordinate system and then (ii) register and resize the head/brain model into the same ovoid 10-20 space.

## 4 Results

The results of ROC analysis and statistical testing of the simulations are shown in this section. To be concise, we use “known” and “unknown” registration to denote the analysis conditions that the contrast vectors are calculated based on the actual probe registration (known head size and registration error) and average registration (unknown head size and registration error) in the following context. For the same purpose, ROI radius and separation are used to denote the radius of the spherical surfaces defining the ROIs and the distance between the center nodes of them.

### 4.1 Single ROI Analysis

In this section, we examined the performance of the uniform and proposed tapered contrast vector methods to infer changes about a single ROI in the brain. The size of the ROI was varied from 10 to 36 mm. The methods were examined in the case of both ideally known and unknown (errors) in the probe registration model.

**Fig. 7:**
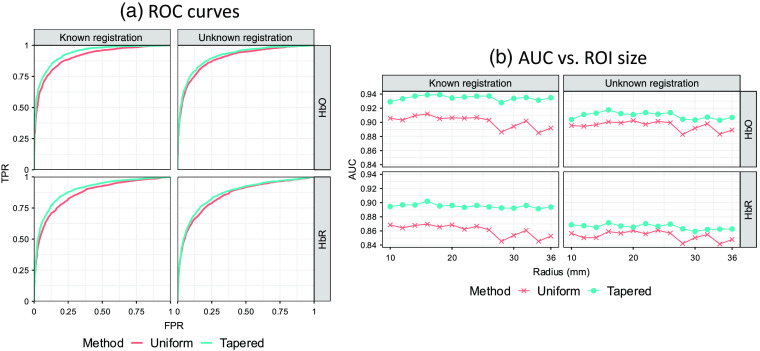
Comparison of analysis methods with uniform and tapered contrast vector using ROC. (a) Each subplot shows the ROC curves of recognizing the hemoglobin activity within a single ROI using the two types of contrast vectors (indicated by color) for data with ROI radius = 14 mm from 2000 iterations of simulations under the conditions where the probe registration information (including head size and registration error) is known or not (indicated by column). The two rows indicate oxy-/deoxyhemoglobin, respectively. (b) Each subplot shows the ROC AUC as a function of ROI radius. Every data point represents the AUC calculated from 2000 simulation iterations against the corresponding ROI radius (in mm) used in the simulation.

Figure 7 is an example of ROC curves of the two analysis methods. The images in Fig. 7(a) demonstrate the full ROC plots for the case of the 14-mm ROI radius. In the case where the probe registration information is known, the AUC for the uniform and tapered contrast vectors, for HbO/HbR, are 0.910/0.868 and 0.937/0.897, respectively, for the 14-mm radius. When an additional registration error is introduced and unknown as described in Sec. 3.1.3, the AUC values are 0.897/0.850 and 0.913/0.865. In both the known and unknown cases, the AUCs are statistically smaller ($p<10^{-5}$) for the uniform compared to the tapered contrast vector. Figure 7(b) shows the AUC as a function of the ROI radius, in which the AUC values were fairly consistent across the tested ROI radius sizes. The tapered contrast vector performs consistently better under all conditions than those with the uniform contrast vector. We also observed that the method using the uniform contrast vector demonstrates more fluctuation when the ROI radius is greater than 28 mm. We believed that this is caused by the gyrus and sulcus since this size is close to the thickness of gyrus and depth of sulcus. In this case, the ROI would include multiple gyri, the space between which may reduce the statistical power of the analyses. Since the contrast vector for a weighted channel is calculated based on the forward model, it already takes the anatomy into consideration and consequently reduces the AUC fluctuation for large ROIs. Statistical tests for the AUC difference between the analyses using the two types of contrast vectors are performed for each simulation ROI radius, i.e., 56 tests (14 radius values × known/unknown conditions × HbO/HBR) are conducted in total. The p-values of the tests for the 28 AUC differences knowing the registration error are all less than $10^{-6}$, which implies that the proposed method performs significantly better than the uniform contrast vector method under this condition. For the unknown condition, although the AUCs of the tapered contrast vector method decrease compared to that when registration information is known, most of the 28 p-values of the tests for the difference between the two methods are smaller than 0.05. The only exception is the AUC for HbR analysis with an ROI radius of 20 mm, the p-value for which is 0.109. It exceeds the commonly used significance level 0.05, however, it is just slightly larger than 0.1. It can still be concluded that the proposed method performs significantly better than the conventional uniform contrast vector method no matter whether the head size and probe registration error is known or not.

**Fig. 8:**
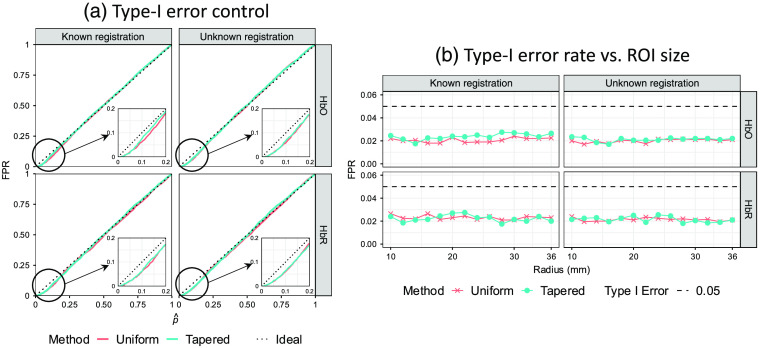
Comparison of analysis methods with uniform and tapered contrast vectors using type-I error control. (a) Each subplot shows the empirical FPR versus reported p-value curves of recognizing the hemoglobin activity within a single ROI using the two types of contrast vector (indicated by color) for data with ROI radius=14  mm from 2000 iterations of simulations under the conditions where the probe registration information (including head size and registration error) is known or not (indicated by column). The two rows indicate oxy-/deoxyhemoglobin, respectively. In an ideal situation, the empirical FPR equals the model-reported p-value, which is represented by the dotted diagonal of each plot. Both methods underestimate the FPR at smaller p-values (enlarged and embedded at the corner). (b) Each subplot shows the ROC AUC as a function of ROI radius. Every data point represents the AUC calculated from 2000 simulation iterations against the corresponding ROI radius (in mm) used in the simulation. Every data point represents the empirical FPR calculated from 2000 simulation iterations against the corresponding ROI radius (in mm) used in the simulation. Here, 0.05 is used as the type-I error control (threshold) that is indicated by the dashed line.

In addition to examining the performance of the ROC analysis with the AUC, we examined the control of the type-I error by comparing the empirically determined FPR with the theoretical values (denoted as p^ [p-hat]). Mismatch between the FPR and p^ indicates either over- or underestimation of the true significance of the results. Figure 8(a) shows the results of the plot of the FPR versus p^ for the simulation with an ROI radius of 14 mm. It can be seen from Fig. 8(a) that the empirical curves are both below the ideal one at the beginning part where FPR and p^ are small. However, they do not remarkably deviate from the ideal curve, so we do not think it is a serious problem. Figure 8(b) shows the empirical FPR for the two analysis methods calculated from simulations with different ROI sizes under the commonly used type-I error control, i.e., significance level α, of 0.05. All data points lay below the dotted line, which indicates that these two methods both underestimate the type-I error with a similar performance. Although type-I error is underestimated by both methods for all ROI radii, we also checked every empirical curve to be sure the p-values are still generally evenly distributed [similar to Fig. 8(a)]. One usually worries about the underestimation of type-I error because it may result in an overestimated type-II error and consequently affect the ROC performance. However, the large AUCs demonstrate the good performances of both methods under all conditions. Thus, we believe the concern for the underestimation of type-I error at small p^ is unnecessary.

### 4.2 Comparison of Two ROIs

In addition to testing the null involvement of a specific ROI, our proposed approach can be used to compare multiple ROIs to each other. In order to examine this, we performed a series of simulations as previously outlined. In addition to varying the location, ROI radius, and probe type, to compare two regions, we also varied the distance between the two regions to examine the limits of this approach. Similar to the characterization of the single ROIs, we preformed simulations to quantify the sensitivity and specificity of the approach in comparison to the use of a fixed and uninform ROI.

**Fig. 9:**
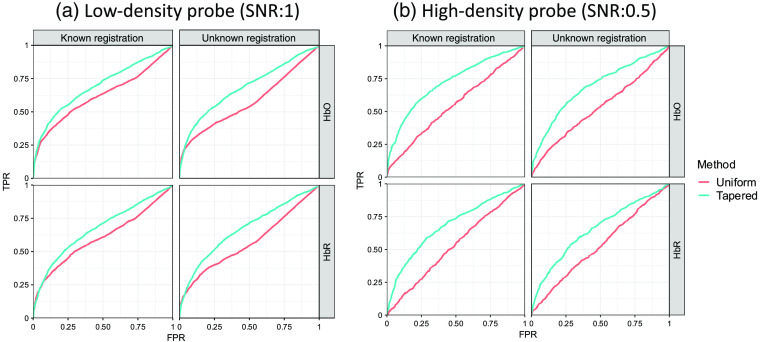
Each subplot shows the ROC curves of recognizing the hemoglobin activity difference between two ROIs using the two types of contrast vectors (indicated by color) for data from 2000 iterations of simulations under the conditions where the probe registration information (including head size and registration error) is known or not. The simulated activities are generated within one of the two 10-mm (radius) ROIs separated by 80 mm, and two types of probe, (a) low-density and (b) high-density, are used in the simulation. The column and row of each subplot indicate known/unknown probe registration and oxy-/deoxyhemoglobin, respectively.

Figure 9 is an example of ROC curves of the two analysis methods for both the low-density and high-density probes, in which 10 and 80 mm are used as the ROI radius and separation, respectively. For the low-density probe, the AUCs of the two methods, uniform and proposed tapered contrast vector for HbO/HbR when the probe registration information is known are 0.622/0.605 and 0.696/0.673, respectively, and the values when the probe registration information is unknown are 0.576/0.570 and 0.684/0.671. For the high-density probe, these values were 0.550/0.529 and 0.724/0.685 for the known probe registration case and are 0.531/0.530 and 0.691/0.654 for the unknown case. In all comparisons, the tapered contrast vector approach preformed statistically better ($p<10^{-5}$) than the uniform weighing approach.

**Fig. 10:**
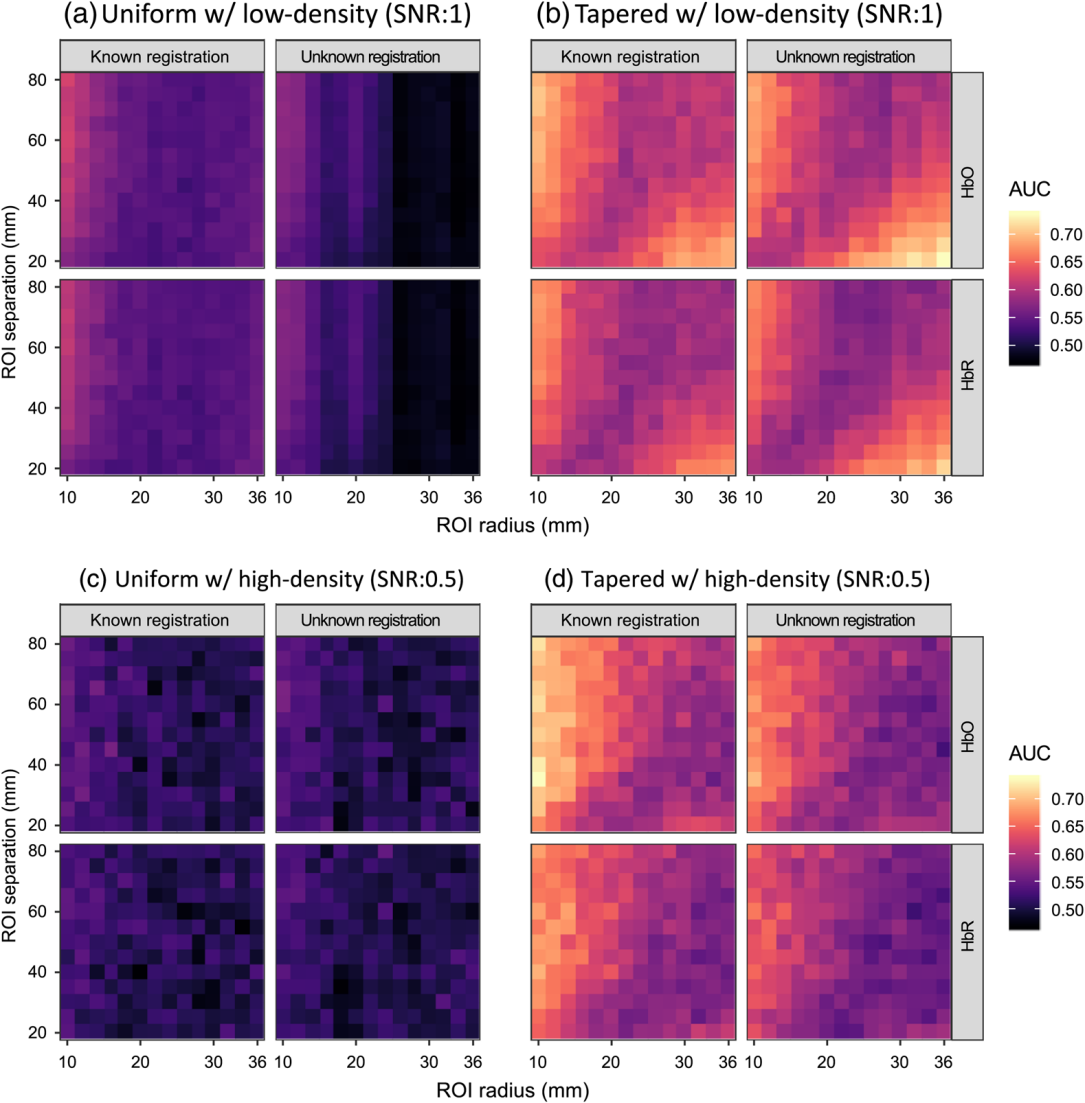
The heatmap showing the AUCs of recognizing the hemoglobin activity difference between two ROIs for simulation data under the conditions where the probe registration information is known or not. The color of each small rectangle in the lattices, whose scale is indicated by the legend, represents the AUC calculated from 2000 simulation iterations using its abscissa and ordinate as the ROI radius and separation, respectively. Two types of probes, (a), (b) low-density and (c), (d) high-density, are used in the simulations, and the results using (a), (c) uniform contrast vector and (b), (d) tapered contrast vector, respectively. Within each panel, the column and row of each subplot indicate known/unknown probe registration and oxy-/deoxyhemoglobin, respectively.

Figure 10 shows the AUC of the two methods for the ROI difference analysis by varying the ROI size and separation in the simulation, from which we can see that the analysis using the spatially tapered contrast vector performs consistently better than that using uniform contrast vector under all conditions since its AUCs are in a higher color range. By performing statistical tests for the AUC differences between the two methods for the simulations using low-density probe, i.e., comparing each pair of small colored rectangles at a corresponding position in the lattices of Figs. 10(a) and 10(b), we obtained significant p-values (smaller than 0.05) for each pair of AUCs using tapered and uniform contrast vector across all ROI radii, separation distances, and analysis conditions. The maximum p-values is 0.0257 for the AUCs comparing the HbR changes within two ROIs with 16-mm radius separated by 25 mm knowing the probe registration error. Similar tests are conducted for the high-density probe simulations, i.e., Figs. 10(c) and 10(d), in which the all obtained p-values are smaller than 0.05, with uniform contrast vector across all ROI radii, separation distances, and analysis conditions. The maximum p-value is 0.0250 for the AUCs comparing the HbO changes within two ROIs with 36-mm radius separated by 80 mm without knowing the probe registration error. We can conclude that the proposed method performs significantly better than the conventional methods for the ROI difference test using both low- and high-density probes.

**Fig. 11:**
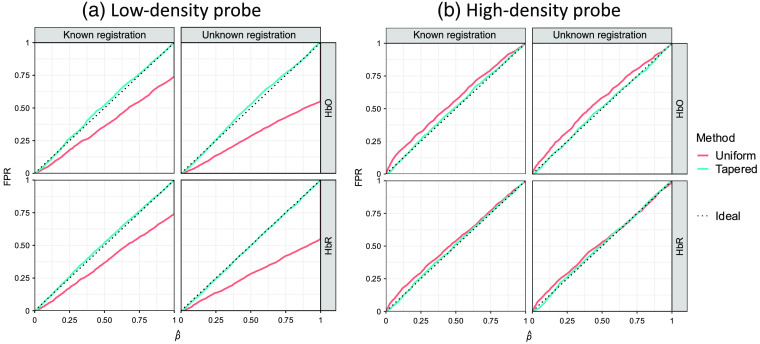
Each subplot shows the empirical FPR versus reported p-value curves of recognizing the hemoglobin activity difference between two ROIs using the two types of contrast vectors (indicated by color) for simulation data analyses under the conditions where the probe registration information is known or not. The simulated activities are generated within one of the two 10-mm (radius) ROIs separated by 80 mm, and two types of probe, (a) low-density and (b) high-density, are used in the simulation. The column and row of each subplot indicate known/unknown probe registration and oxy-/deoxyemoglobin, respectively. For a single curve, the abscissa of each data point is the FPR using its ordinate as the threshold. In an ideal situation, the empirical FPR equals the model-reported p-value, which is represented by the dotted diagonal of each plot.

Finally, we examined the type-I error control for the comparison of two ROIs using the tapered and uniform approaches. In Fig. 11, we demonstrate these results for simulations using ROI radius of 10 mm and separation of 80 mm. In comparison to the single ROI analysis (shown in Fig. 8), we found that using uniform contrast vector consistently underestimates the FPR in the case of the low-density probe. It can be seen from Fig. 11(a) that the p^ reaches 1 where the FPRs using uniform contrast vector are only 0.55 and 0.74 when the probe registration is known and unknown, respectively. This means that in 45% and 26% of cases, this method is not able to distinguish between the two ROIs. The reason is that the uniform contrast vectors for the two ROIs can be exactly the same when the two ROIs are close enough to each other, which results in an all-zero contrast vector for the ROI difference test and consequently a zero t-statistic giving a unity p-value. In the case of using a tapered contrast vector, the two contrast vectors will never be the same no matter how close they are as long as they are not completely overlapping each other. Therefore, the p^ reported by the tapered contrast vector method appropriately estimates the empirical FPR, which demonstrates this method has a higher spatial resolution than the other two. This also explains why the ROC curves of uniform contrast vector-based method achieve diagonals at 0.55 and 0.74 in Fig. 9(a). This is why we investigated this problem again using a high-density probe that has a higher spatial resolution and is expected to improve the type-I error rate with the uniform contrast vector. For the high-density probe [Fig. 11(b)], the type-I error is slightly underestimated for the uniformly weighted model, which results in increased false positives. However, the two ROIs are more distinguishable. In both probes, the proposed tapered contrast vector appropriately estimates the FPR.

**Fig. 12:**
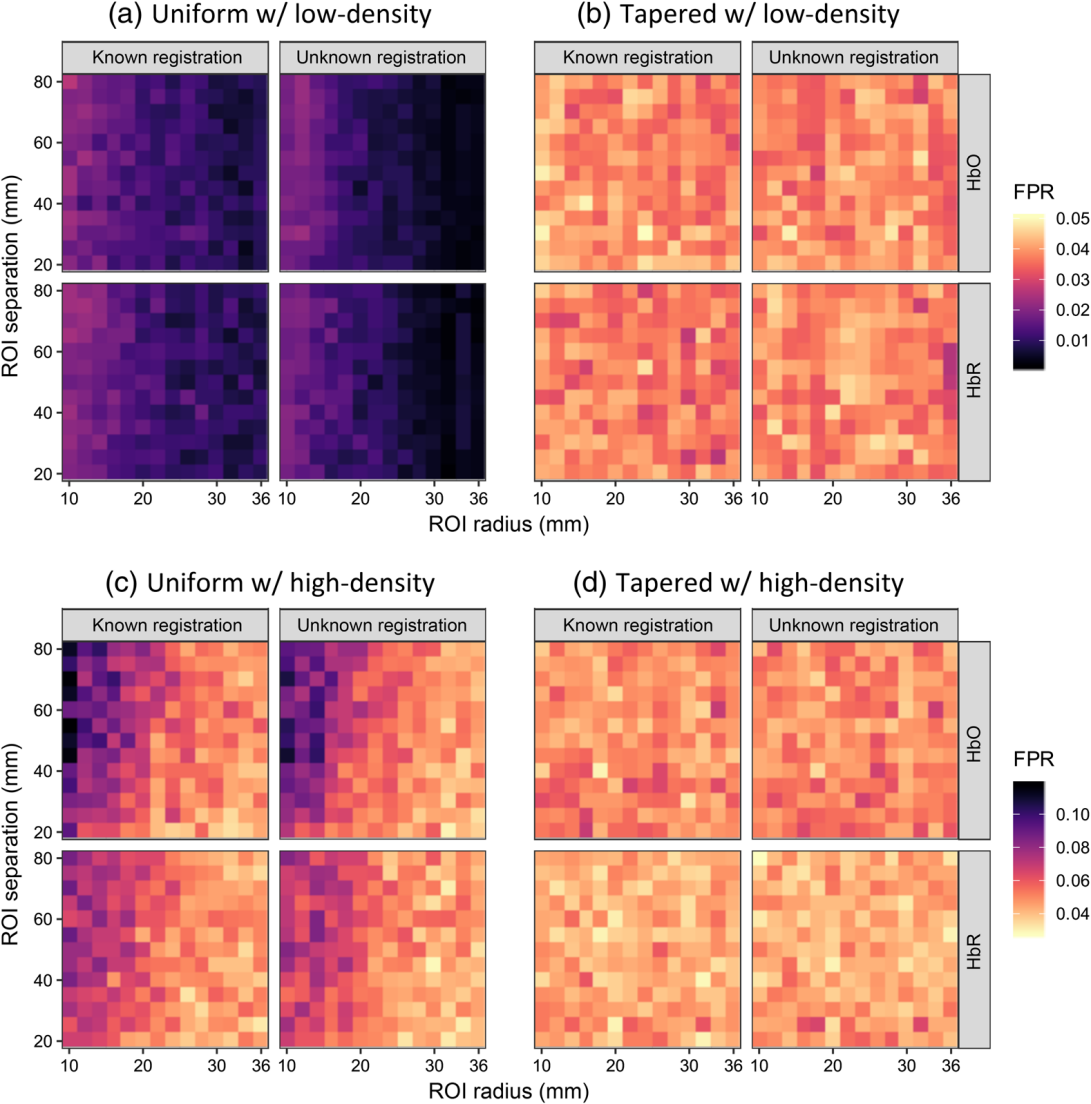
The heatmap showing the empirical FPRs of recognizing the hemoglobin activity difference between two ROIs using the two types of contrast vector for simulation data analyses under the conditions where the probe registration information is known or not. The color of each small rectangle in the lattices, whose scale is indicated by the legend, represents the FPR calculated from 2000 simulation iterations using its abscissa and ordinate as the ROI radius and separation, respectively. Two types of probe, (a), (b) low-density and (c), (d) high-density, are used in the simulations, and the results using (a), (c) uniform contrast vector and (b), (d) tapered contrast vector. Within each panel, the column and row of each subplot indicate known/unknown probe registration and oxy-/deoxyhemoglobin, respectively. Here, 0.05 is used as the type-I error control (threshold) that is indicated by the bright color.

In Fig. 12, the FPR at p^=0.05 is shown for various ROI radii and separation distances. The four panels represent the same analyses as those in Fig. 10. The colors in the heatmap of the tapered contrast vector method, i.e., Figs. 12(b) and 12(d), fall into the range around 0.05 with both low-and high-density probes, which indicates the FPR estimation is generally appropriate. For the uniform contrast vector method, i.e., Figs. 12(a) and 12(c), the colors are completely out of the appropriate range when the low-density probe is used. For a given ROI separation distance, the FPRs reported by the uniform contrast vector method decrease and deviate further from the type-I error control p^=0.05 because the enlarging overlap of the two ROIs makes it more difficult to distinguish between the two ROIs. For the case of using the high-density probe, although most of the colors are in an appropriate range and it can still be seen that the type-I error rate is overestimated for small ROIs with large separations [note that darker color represents a larger value in Figs. 12(c) and 12(d)]. However, the plots of the worst case (10-mm radius and 80-mm separation) have been shown in Fig. 11(b), from which we can see that the empirical curves do not significantly deviate from the ideal curve.

## 5 Discussion and Conclusion

In this paper, we show the analysis results of thousands of simulations using 2 (probe layouts) × 14 (radius lengths) × 13 (separation distances) = 364 parameter combinations. Here, we discuss the findings and draw conclusions in the following aspects.

### 5.1 Comparison of Multiple ROIs

It can be seen from Fig. 10 that the two factors, ROI radius and separation, jointly affect the results. The effects of channel selection, uncovered area, and blind-spot can be different with different ROI radii and separations. In general, the channels selected for calculating contrast vectors of the two ROIs tend to be same as the two ROIs get larger and closer and larger ROIs with a greater separation are more likely to have a larger portion exceeding the probe coverage, while smaller ROIs are easier to fall into the blind-spot of the probe. The statistical power and AUC will reduce under these three conditions. We will explain the AUC changes in terms of these three effects. Let us first look at the heatmaps of the analysis using the uniform contrast vector with known registration information in the left column of panel (a). With this analysis method, the AUC increases as the two ROIs are separated by a greater distance given a specific ROI radius and decreases as the radii of ROIs increase given a specific ROI separation when the registration is known. This pattern is not difficult to understand. Since the nearest four channels are used with equal weights, the contrast vectors of smaller ROIs with larger separations will have fewer shared channels, and the contrast vector for their difference is further from 0, resulting in a more significant t-statistic/p-value and vice versa. Another negative effect of a large ROI is that ROIs with larger radii are easier to partially fall out of the probe coverage area, especially for further separated ROIs, as they are more likely to be close to the edge of the probe, which reduces the statistical power when a stimulus exists within the ROI. These are the reasons that the AUCs are larger for the small-radius large-separation condition. For the condition using the uniform contrast vector without knowing the registration in the right column of panel (a), the AUC still increases as ROI separation increases given a specific ROI radius for the same reason, while the decreasing pattern of the AUC along the ROI radius does not always hold. There is an increase in AUC when the ROI radius is around 20 mm. Unlike the analysis when given the registration error, here, the four channels used for contrast vector generation are selected based on the Colin27 atlas with an average head size and no registration error. The selected channels can be different from the nearest four channels in truth if there is a large enough difference in the head size or probe registration between the subject probe and the average probe. Thus, the channel selection error is another factor affecting the analysis using this method. For a fixed probe registration difference including both effects of head-size difference and registration error, the relative registration difference for a smaller ROI will be larger than that for a larger one, which means that the possibility of using the wrong channels for contrast vector calculation of smaller ROIs is higher. Although the contrast vector of the difference between two larger ROIs has a negative effect on the AUC (as explained for the known registration condition), the possibility of using wrong channels for larger ROIs is reduced. Hence, the AUC change against the ROI radius for a specific ROI separation is nonmonotonic. This is the reason we see a sudden increase in AUC around ROI radius=20  mm. The change of AUCs using the tapered contrast vector is more complicated. We can see that the increasing/decreasing pattern found for uniform contrast vector analysis is only true in the upper triangle whereas an opposite pattern appears in the lower triangle of subplot (b). The effects of factors affecting the AUCs using a uniform contrast vector analysis still hold for the analysis with a tapered contrast vector. However, the difference between the two tapered contrast vectors is further from 0 than that of the uniform contrast vectors, especially for closer and larger ROIs that are more likely to result in two uniformly weighted contrast vectors that are exactly the same. This implies that the AUC decrease caused by the contrast vector decrease is smaller than the uniform method for closer and larger ROIs (the lower triangle area). Specifically, (i) larger ROIs with a given separation are more likely to have uncovered area by the probe, so the effect of decreasing this possibility dominates that of smaller contrast vector, as explained before, with the decrease in ROI separation; (ii) closer ROIs with a fixed size are expected to have smaller uncovered areas by the probe, and smaller ROIs are more likely to fall into the “blind-spots,” so the effect of decreasing this possibility dominates that of the uncovered and smaller contrast vector with the increase in ROI size. The increase in AUC can be consequently seen as an ROI separation decreases and the ROI radius increases for large ROIs with a smaller separation. These are the reasons that we see an opposite pattern in the lower triangle area of the plot.

### 5.2 Effect of Probe Registration Errors

To evaluate the effect of using probe registration errors in the analysis, we compared the results of analyses with known or unknown registration errors for all simulation parameters and conditions. In the single ROI analysis, we can see from Fig. 7(b) that the AUCs of both tapered and uniform methods are improved with the registration errors provided (left two subplots) comparing to the analysis without knowing the errors (right two subplots). It can also be found that the improvements of tapered contrast method are larger. We also conducted statistical tests on the significance of these improvements, from which significant p-values (smaller than 0.05) are reported for all of the improvements using a tapered contrast vector, but the p-values are only significant for small ROIs (radius<30) using the uniform contrast vector. This means that no significant improvements are found for large ROIs when the uniform contrast vector is used. As explained in Sec. 5.1, there is a possibility that the four channels identified without registrations are different from the nearest four channels in the truth. In the single ROI analysis, the information of registration errors can help with determining the correct four channels when using a uniform contrast vector. However, the possibility of choosing the wrong channels for larger ROIs is smaller compared to that for smaller ROIs. This explains why the improvements for large ROIs are insignificant. For the tapered contrast vector, the registration errors can correct the calculation of the weights in the contrast vector. The contrast vectors calculated with and without registration errors can never be the same regardless the size of the ROI. Thus, using the registration errors always significantly improves the ROC performance of the tapered contrast vector. In the comparison of two ROIs, the analyses using the registration errors also improve the AUCs compared to those without the errors. We performed similar statistical testing between the AUCs with and without knowing the registration errors. However, only about 30% of the tests report significant p-values for both uniform and tapered contrast vectors, and the appearance of these p-values shows a random pattern, which does not make much sense to discuss. In summary, utilizing the information of registration errors can improve the analysis performance, especially cooperating with the tapered contrast vector in single ROI analysis.

### 5.3 Probe Comparison

The effects of factors affecting the AUCs of the low-density probe, as explained in Sec. 5.1, still hold for the high-density probe [Figs. 10(c) and 10(d)]. For the analyses using a uniform contrast vector, although we can see a similar changing pattern to the one shown in Fig. 10(a), the AUCs do not notably change with the change in ROI radius. The reason for this is that the possibility of obtaining an all-zero contrast vector high density is rare unless the two ROIs are close enough, since many more channels are used to construct the uniform contrast vector compared to the low-density probe. Hence, the detrimental effect of large ROIs on the contrast vector is reduced. For the tapered contrast vector, the AUC changing pattern is also similar to Fig. 10(b) except that the AUC rise for large-size small-separated ROIs is smaller. This is because the high-density probe also reduces the number and size of “blind-spots” and the possibility of small ROIs falling into “blind-spots” is smaller than that with low-density probe, i.e., the negative effect of “blind-spots” is reduced. Thus, although increasing the radius for short-separated ROIs can get rid of the effect of “blind-spots,” this effect itself is smaller and so is the AUC rise.

It might be noted that the AUCs using the high-density probe do not show a remarkable improvement compared to that using the low-density probe. This is because a different SNR is used for the high-density probe, which is indicated in the title of each panel. The SNRs used in the ROC simulations were chosen to generate nontrivial comparisons of the methods being tested (e.g., with too high an SNR, all methods converge on AUC=1, while with too low an SNR, all methods approach chance levels). In practice, one is expected to see an improvement when switching to the high-density probe from a low-density probe for the same experiments. Due to the same reason, it is impossible to conduct direct statistical testing between the performances of the two probes.

### 5.4 Robustness of the Analysis

The analyses conducted in this study demonstrate that the proposed method constructs a channel-space statistic that can be used to statistically test the noninvolvement of a specific ROI and the activity difference between two ROIs during a functional task utilizing the optical forward model as the channel weights without solving the underdetermined ill-posed image reconstruction inverse model. Although the computation of the tapered contrast vector depends on many factors including the forward model approximation, wavelength, brain anatomy, etc., the differences in these factors do not remarkably change the tapered shape of the contrast vector. Moreover, we also check the difference between different forward models as well as the contrast vectors calculated using various wavelengths. The computation shows the correlations between the forward models generated via the slab approximation and using NIRFAST is 0.921, the error between which is around $1-0.921^{2}=15\%$, and the change in wavelengths between 660 and 890 nm only makes a 4.6% difference in the contrast vector magnitude. Thus, the computation precision using the ApproxSlab forward model and an 808-nm wavelength is acceptable. Introducing the complexity of the forward model approximation and wavelength will not notably change the analysis results.

### 5.5 Comparison of Uniform and Tapered Weighting Methods and Overall Recommendations

Going through all the results shown above, we can conclude that the proposed tapered contrast vector performs better than the conventional uniform one. In terms of ROC performance, its AUC is significantly larger than the conventional method for both single ROI analysis and two-ROI comparison regardless of ROI size, separation distance, and probe layout selection. The p-values for the difference between the AUCs are smaller than 0.05 with only one exception, which is slightly larger than 0.1. For the type-I error control, both methods are generally appropriate with the low-density probe in a single ROI analysis, although the type-I error rates are underestimated at the commonly used threshold of 0.05. However, in the comparison of two ROIs, the proposed tapered contrast vector method always appropriately estimates the type-I error while the conventional method always underestimates and sometimes overestimates the type-I error rate when the low- and high-density probes are used. In conclusion, the proposed tapered contrast vector is always recommended for ROI-based analysis.

### 5.6 Limitations and Future Plan

Although this work demonstrates that the proposed method is significantly better than the conventionally used method, it still has several limitations. First, in the single ROI analysis, the type-I error rate is underestimated at the widely used significance level, i.e., 0.05. Although using a high-density probe could solve this problem, considering the small improvement space in AUC and the time and cost consumption of a high-density probe, we do not think it is worth using high-density probe in this problem. Second, the performance for the comparison of two ROIs is not good enough. There is still a space for ROC AUC improvement. Third, the model is still based on a misregistered probe when the registration information is unknown and the anatomical difference between subjects is not involved.

Therefore, the next step of this work will include introducing anatomy variations and optimizing probes based on an image reconstruction model considering the probe is a random factor that deviates from an optimal average probe position. It is reasonable to believe that the tapered contrast vector calculated based on the optimal probe would provide a better analysis.
